# Circumscribed Traumatic Aneurism of the Left Femoral Artery—Ligation of the External Iliac Artery—Gangrene of Leg, and Death on the 40th Day

**Published:** 1894-01

**Authors:** Howard J. Williams

**Affiliations:** Macon, Ga.


### Southern Medical Record.

A MONTHLY JOURNAL OF MEDICINE AND SURGERY.

VOL. XXIV. ATLANTA, GA., JANUARY, 1894. No. 1.

### Original Articles.

### CIRCUMSCRIBED TRAUMATIC ANEURISM OF THE LEFT FEMORAL ARTERY—LIGATION OF THE EXTERNAL ILIAC ARTERY—GANGRENE OF THE LEG, AND DEATH ON THE 40th DAY.

BY HOWARD J. WILLIAMS, A. M., M. D., MACON, GA.

The following report was written for the Medical Association of Georgia, in 1892, but owing to my inability to attend the meeting at Columbus, the notes were put away with the purpose of publishing them later. They have remained unpublished until a few days ago, when I decided that even at this late day I would publish them, as the case is one of interest to myself and perhaps will be so to others.

Dolly B., negro female, aged 15 years, received in February, 1891, (exact date not remembered by her friends) a pistol shot wound, No. 38, in the left thigh. The ball entered the middle of the groin, one and one-half inches below Pouparts Ligament, and made its exit posteriorly and interiorly four inches above the knee joint. At the time of the accident there was very little hemorrhage. Soon after the accident, however, a pulsating tumor of gradual growth developed between the two external wounds. This tumor continued to grow until I saw the patient, August 25th, 1891, by request of Dr. N. G.

Gewinner, who was then attending the case. At that time it was as large as a man's head, extending from Poupart's Ligament down to the middle of the thigh. The skin over it was tense and glistening, and the tumor was extremely painful on manipulation and on motion. Pressure over the region of the External Iliac Artery controlled the pulsation and diminished the bruit on auscultation. The limb below the tumor was œdematous, numb and painful only on motion, and sensation on pricking the skin was indistinctly felt. The general condition of the patient extremely low, wasted and anaemic, temperature 100:5 ; pulse 136 ; sleepless and nervous, owing to extreme pain.

Owing to the unhygienic surroundings of her home, and the inability of her parents to take proper care of and nurse her, she was sent to the county hospital ; tonics, stimulants and a generous diet prescribed and an immediate operation advised.

August 29th, 1891, assisted by Drs. McHatton and Derry, and in the presence of Drs. Gewinner, Holt, Gilmer, Heard, K. Hall, and Worsham, I proceeded to ligate the External Ili Artery. After exposing the artery in the usual manner, I placed two large cat-gut ligatures around the artery, one fourth of an inch apart, and closed the external wound with cat-gut sutures. The operation was conducted under thorough antiseptic methods. The patient bore the operation poorly ; free use of whiskey, stychina, and nitro-glycerine hypodemically, and external warmth being required to prevent her from collapsing.

August 30th. Tumor slightly diminished in size ; no pulsation ; no pain ; sensibility to touch acutely present in the thigh, but very slightly felt below the knee. Toes appeared dry and stiff ; some sensation on pricking the sole of the foot. Two large blisters on the calf of the leg, one externally the other internally, attributed to prolonged contact of bottles of very hot water. Temperature 99–99:5 ; pulse 136-144; morning and evening respectively.

September 2nd. Discoloration of lower part of calf of the leg and foot, and blebs formed on the top of the foot ; sensation acute in the upper part of the calf, very indistinct in the lower part of the calf and foot. General temperature 100:6; pulse 136; local temperature below the knee and on the foot 98:6.

September 5th. Eight day after the operation; removed the dressings from the wound in the groin and found that primary union had taken place; the sutures had been absorbed and the line of incision was marked only by a red line of new tissue. No pulsation could be felt over the tumor, which had decreased somewhat in size. Dr. McHatton and I thought we could detect feble pulsation in the Posterior Tibial Artery behind the ankle. The toes were shriveled and had turned dark. The foot and lower part of the calf still discolored and numb, though the local temperature of the parts remained normal. The patient's general condition had improved somewhat since the operation, under iron, quinine and whiskey; appetite good; slept well; temperature 98:6; pulse 120.

September 8th. Discoloration of the calf extended up posteriorly and had reached the popliteal space. Sensation lost on the posterior but present on the anterior surface of the knee. Temperature 99; pulse 125.

September 16th. Gangrene rapidly extended up the limb; surface of inside of the thigh blistered and discharged a thin sanious fluid. Temperature 102:6; pulse 130; chill; general condition very low.

September 17th to October 1st. For unavoidable reasons I was compelled to be away from the city. Drs. McHatton and Derry took charge of the case during my absence. It was their purpose to amputate as soon as the line of demarkation should form. The record showed a continuous temperature of 102 to 103 from September 17th to September 25th, then a gradual fall to 99:4 on the 29th, pulse 136 to 144 with a corresponding depression of the general condition.

October 2nd. Having again taken charge of the case, I found the line of demarkation had completely formed. It extended from the middle of the thigh anteriorly up to the gluteal fold posteriorly. Her condition had somewhat improved. Temperature 99, pulse 126. The possibility of a high amputation or at the hip joint, presented itself to us, and we decided to attempt amputation the next morning, (October 3rd) if the patient's condition at that time would warrant the risk. During the night, however, she had several convulsions, the temperature had risen to 105, pulse 150. Nothing further could be done for the case, except to control the convulsion by anodynes. In this condition the girl lived, having convulsions at intervals of one to three hours, for four days, dying October 7th.

Should I ever be called upon to operate in a similar case, I will not perform the above operation, but will cut down into the aneurismal sac and secure the ends of the injured vessel above the aperture in the artery. I believe this would give a better chance for the early restoration of collateral circulation, and possibly the limb and life of the patient. Owing, however, to the extreme exhaustion of the present case, we feared our patient could not survive the more hazardous operation of cutting into the sac and tying the ends of the artery, and in consultation decided upon Hunter's operation. Hunter's operation will do for spontaneous aneurisms or for circumscribed traumatic aneurisms of arteries lower down the limb. In 1890, I ligated the Femoral at Hunter's Canal for circumscribed traumatic aneurism of the Tibial Artery high up in the calf (Atlanta Medical and Surgical Journal, March 1891) with complete success. In that case no untoward results followed owing to the early establishment of collateral circulation.

